# Predictive Modeling of a Leaf Conceptual Midpoint Quasi-Color (CMQ) Using an Artificial Neural Network

**DOI:** 10.3390/s20143938

**Published:** 2020-07-15

**Authors:** Ivan Simko

**Affiliations:** U.S. Department of Agriculture, Agricultural Research Service, U.S. Agricultural Research Station, Crop Improvement and Protection Research Unit, Salinas, CA 93906, USA; ivan.simko@usda.gov

**Keywords:** anthocyanins, artificial neural network, chlorophylls, leaf color, plant pigments, predictive modeling

## Abstract

The color of plant leaves is moderated by the content of pigments, which can show considerable dorsiventral distribution. Two typical examples are leafy vegetables and ornamentals, wherein red and green color surfaces can be seen on the same leaf. The proof of concept is provided for predictive modeling of a leaf conceptual mid-point quasi-color (CMQ) from the content of pigments. The CMQ idea is based on the hypothesis that the content of pigments in leaves is associated with the combined color from both surfaces. The CMQ, which is calculated from CIELab color coordinates at adaxial and abaxial antipodes, is thus not an actual color, but a notion that can be used in modeling. The CMQ coordinates, predicted from the content of chlorophylls and anthocyanins by means of an artificial neural network (ANN), matched well with the CMQ coordinates empirically found on photosynthetically active leaves of lettuce (*Lactuca sativa* L.), but also with other plant species with comparable leaf attributes. Modeled values of lightness (qL*) decreased with the increasing content of both pigments, while the redness or greenness (qa*) and yellowness or blueness (qb*) of the CMQ were affected more by a relative content of chlorophylls and anthocyanins in leaves. The highest vividness of quasi-colors (qC*) was modeled for leaves with a high content of either pigment alone. The model predicted a substantially duller quasi-color for leaves with chlorophylls and anthocyanins present together, particularly when both pigments were present at very high levels.

## 1. Introduction

The pigments in plant leaves play a critical role in biological functions, such as capturing light energy for photosynthesis or mitigating stresses caused by biotic and abiotic factors [1]. The amount and combination of pigments in leaves affects visual perception of their color, the trait that is vital to consumers of leafy vegetables and growers of ornamental plants. Moreover, plant pigments have a beneficial effect on human health [1], making them a highly desirable target of plant breeding programs. Plant phenotyping is a rapidly growing research area that utilizes sensors to nondestructively monitor plant traits [2,3], including evaluations of leaf color and leaf pigment content that can provide helpful insight into the physiological performance of leaves [4]. The three basic types of pigments that cause leaf coloration are chlorophylls (green color), anthocyanins (red–purple color) and carotenoids (yellow–orange color), though the bright color of carotenoids is usually masked in photosynthetically active tissues and revealed only after the degradation of chlorophylls occurs [5]. Combinations of these plant pigments were used for modeling virtual [6,7,8,9,10] or real colors of fruits, vegetables [11], leaves [12], and the mechanism of color change [13]. For example, the color of leaves and its change were modeled using simulated concentrations of chlorophylls, carotenes and anthocyanins [10], or actual concentrations of chlorophylls and carotenes [7]. In simple systems, where only one pigment is predominant, a strong relationship between concentrations of this pigment and color may be observed [11], e.g., between chlorophylls and a green color [9,14] or between anthocyanins and red a color [15,16]. However, in more complex systems, wherein multiple pigments occur within the same tissue, these simple relationships cease to be visible [11] due to spectral inference between pigments [4].

Leaf color can be defined using the CIELab color space that approximates human visual perception [17]. This color space is characterized by three coordinates: L*, a* and b*. L* indicates lightness of color and ranges from 0 (minimum, darkest black) to 100 (maximum, brightest white), a* indicates redness (positive values) or greenness (negative values), while b* indicates yellowness (positive values) or blueness (negative values) of color. The combination of a* and b* coordinates provides information about chroma (C* = $\sqrt{a^{*}{}^{2}+b^{*}{}^{2}}$) and hue angle (*h°* = $arctan\;\left(\frac{b^{*}}{a^{*}}\right)$). Chroma describes vividness of the color, with higher values indicating more vivid (less dull) color, while the hue angle is associated with the dominant color as perceived by an observer. The hue angle values are usually defined as red–purple at 0°, yellow at 90°, bluish-green at 180°, and blue at 270°. The difference between two colors (Δ*E*) can be calculated from changes at each of the three coordinates as: $∆E=\sqrt{∆L^{2}+∆a^{2}+∆b^{2}}$.

CIELab or other color space (e.g., RGB) coordinates were previously corelated with the content of either chlorophylls [9,14] or anthocyanins [15,16], but were not investigated on leaves with varying contents of both pigments combined. Moreover, these analyses were performed using statistical models with restrictive structural assumptions. It is also important to note that most of the published associations between leaf color and color coordinates were performed on only a single leaf surface (usually adaxial) [14,15], though a large number of plant species have bifacial leaves that show substantial dorsiventral distribution of pigments. Lettuce, the most popular commercially produced leafy vegetable [18], is a typical example of diversity in leaf color across cultivars, but also between two leaf surfaces. The color can range from very light green to dark green, and from very light red to dark red, to almost black [19]. These large variations make the modeling of lettuce leaf color challenging because the content of pigments cannot be unambiguously associated with the color on either one of the surfaces.

To assess the association between pigments and the color on leaves showing dorsiventral distribution, the color on both leaf surfaces needs to be considered. It could be hypothesized that the amount of pigments in leaves, particularly in cases where differences in pigment and color distribution between adaxial and abaxial surfaces are substantial, would match more accurately with combined color coordinates from both surfaces, rather than with color coordinates from only one of the surfaces. The combining of color coordinates can be done through the concept of ‘quasi-color’ at ‘mid-point’. Mid-point is a conceptual point between two antipodal points on leaf adaxial and abaxial surfaces, which combines color coordinates from both of them. The conceptual mid-point quasi-color (CMQ) is thus not an actual color of the leaf, but a notion that can be used in modeling. The qL*, qa* and qb* vector coordinates of the CMQ are calculated from two three-dimensional vector coordinates of color at antipodes, as: $(\frac{L_{D}^{*}+L_{B}^{*}}{2}$), $\left(\frac{a_{D}^{*}+a_{B}^{*}}{2}\right)$ and $\left(\frac{b_{D}^{*}+b_{B}^{*}}{2}\right)$, where L*, a* and b* are the color coordinates for antipodal points that are located on adaxial (subscript D) and abaxial (subscript B) surfaces. Quasi-color chromaticity (qC*) and the hue angle (*qh°*) are calculated from qa* and qb* coordinates identically, as in the CIELab color space. The CMQ coordinates can subsequently be used to evaluate association with the content of chlorophylls and anthocyanins in leaves.

The objective of this work was to model CMQ for healthy, photosynthetically active leaves of lettuce, and test how this model applies to leafy vegetables and other plant species. The concentrations of pigments in leaves were used to model expected CMQ coordinates, employing full factorial, quadratic polynomial, and response surface designs. However, because the extent and the relationship of spectral interference between the two pigments is unknown, the modeling was also performed with artificial neural networks (ANN). ANN computing systems, inspired by the biological neural networks of animal brains, have the ability to learn from training data [20] and apply such learned knowledge to mapping the relationships between input and output data. This is done by creating intermediary transformation layers that calculate both the linear and nonlinear relationships between inputs and outputs [21]. Hence, ANN has the ability to capture patterns between data even in non-linear, complex systems [22]. Moreover, its versatility and model-free approach allows the modeling of underlying processes without restrictive assumptions [23]. In plant research, modeling with neural networks has been successfully used in a wide range of applications, including optimizing growth media [24,25], classifying cell wall architecture [26], identifying diseases [27,28], analyzing biophysical properties [29], forecasting pH and electrical conductivity [30,31], evaluating post-harvest changes and product quality [32,33,34], and characterizing and authenticating plant products [35]. Because of the unknown extent of spectral interference between the two pigments, an ANN appears to be a convenient method for modeling CMQ coordinates, using chlorophyll and anthocyanin content as input parameters and qL*, qa*, and qb* values as output parameters.

## 2. Materials and Methods

To predict CMQ from the concentration of pigments, four models were developed and tested on lettuce because its leaves can contain highly variable concentrations of both chlorophylls and anthocyanins. The best-performing model was subsequently applied to samples from additional plant species in order to verify that the model can also be used on other herbaceous leaves similar to lettuce.

### 2.1. Plant Material and Datasets

Lettuce plants of varying color were grown in a greenhouse or in an open field. Evaluations of leaf color and quantifications of pigments were performed on 604 samples from ca. 30–60-day-old plants (primary dataset, Additional file 1 in Supplementary Materials). Because the objective of this study was to develop the CMQ model for photosynthetically active leaves, only leaves containing chlorophylls were investigated. Separately, the contents of pigments and leaf color were determined on another batch of 129 samples (ancillary dataset, Additional file 2 in Supplementary Materials) that comprised lettuce, leafy vegetables and other plant species (collected from nature). This set included also a few yellow-, almost white-, and orange-colored leaves, plus flower bracts and petals that were used to assess the performance and the limitations of the tested models outside of their original scope. Samples did not have any obvious blemishes, substantial wax deposit, trichomes, or other visible features that could affect their color. A thin layer of epicuticular wax or minor trichomes on five samples from the ancillary dataset were removed by gently rubbing their surface with tissue paper. These samples were not outliers in any of the analyzed parameters, therefore they were kept in the dataset. The common and scientific names of plant species were used according to The PLANTS Database [36].

### 2.2. Quantification of Pigments and Measuring Leaf Color

The content of chlorophylls and anthocyanins was determined on leaves using SPAD-502 (Konica Minolta Sensing Inc., Tokyo, Japan) and ACM-200 plus (Opti-Sciences, Hudson, New Hampshire, USA) meters. These devices use light transmittance to provide good in situ estimates of relative contents of the two pigments [37,38]. The content of chlorophylls was measured in SPAD units, while the content of anthocyanins was measured in anthocyanins content index (ACI) units. Measurements of chlorophylls and anthocyanins were taken at antipodal points on adaxial and abaxial leaf surfaces and averaged for each pigment. Measurements of color were performed using RM200QC spectrocolorimeter (X-Rite, Grand Rapids, Michigan, USA) on the same adaxial and abaxial leaf areas as those used for evaluations of chlorophylls and anthocyanins. All measurements of color were done with the D65/10° setting that represents noon daylight with a 10° wide view standard observer. SPAD and ACI units were transformed prior to statistical analyses to improve normality of data distribution. For SPAD data, the best results were achieved using square root transformation, and thus the chlorophylls units are shown as ‘SR-S’ (square root of SPAD). For ACI data, the best results were achieved using logarithmic transformation. To obtain a similar range of transformed data for both pigments, the binary logarithm was applied to transform the ACI values. The transformed anthocyanin units are shown as ‘Lb-A’ (binary logarithm of ACI). To avoid the problem with ‘wraparound’ (crossing 360°) when performing statistical analyses on hue angles (e.g., the mean of 350° and 16° is 3°, not 183°), the angles between 180° and 360° were transformed by subtracting 360°, thus converting the hue scale to the −180° to 180° range. The threshold of 180° was selected because there were no leaves with *h*° at this range (the hue of cyan, teal or blue is rare in leaves). Thus, a value of, e.g., 345° (purple hue) would be entered into statistical analyses as −15°. The −180° to 180° scale was used in all statistical analyses, tables and figures; however, for consistency with CIELab color space, the hue angles were converted back to the original 0° to 360° scale when described in the text.

### 2.3. Parametric Models for Predicting CMQ Coordinates

The parametric models for predicting CMQ coordinates from chlorophyll and anthocyanin content were based on three methodologies: full factorial, quadratic polynomial and response surface. In order to develop respective models and to evaluate their performance, the primary dataset with measurements taken on 604 lettuce leaves was randomly split into the training and testing datasets. Approximately 80% of the data (from 480 leaves) were used for developing models (training dataset) while the remaining ~20% of the data (from 124 leaves) were used to evaluate the quality of the models (testing dataset). The performance of the models was compared using the coefficient of determination (*R*^2^) and the root mean square error (RMSE) calculated between the predicted (modeled) and the observed color coordinates.

### 2.4. ANN Models for Predicting CMQ Coordinates

**Figure 1:**
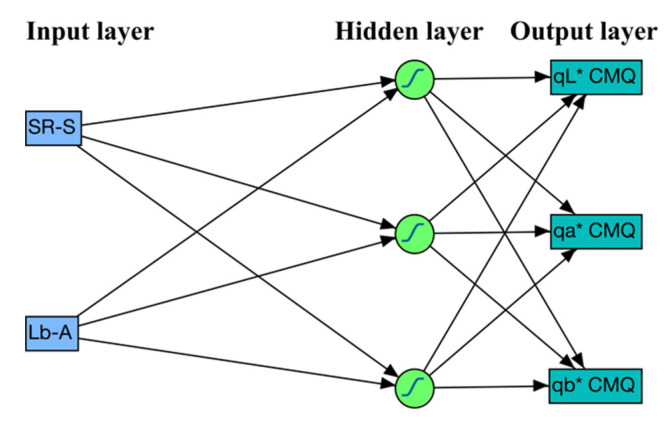
Schematic structure of the selected artificial neural network. The input layer has two nodes (SR-S and Lb-A), the hidden layer has three nodes, and the output layer (response) has three nodes (qL*, qa* and qb*). The hyperbolic tangent (TanH) was used as the transfer function for the hidden layer of nodes.

The primary dataset consisting of data collected from 604 lettuce leaves was used also for the development and evaluation of models based on ANN. An ANN consists of three types of layers, namely, input (initial data), hidden (computational) and output (results). Before an ANN can be used to map the relationship between input (pigments) and output (CMQ coordinates) data, it needs to be trained to do that. The training process was performed on the training set (data from 480 leaves) using the input layer consisting of two nodes (SR-S and Lb-A values) and the output layer consisting from three nodes (qL*, qa* and qb*). The optimum number of hidden nodes in the hidden layer was determined through the K-fold (K = 10) cross-validation approach that can both gauge the quality of the particular ANN model and identify instances when over-fitting of the model has occurred. With K = 10, the training dataset was split into two datasets in a 9:1 ratio. The larger dataset with data collected from 432 lettuce leaves was used to train ANN, while the smaller dataset (validation set) with data from 48 lettuce leaves was used to validate the ANN models. Splitting of the dataset and the cross-validation procedure were performed 20 times for the number of hidden nodes from 1 to 25 (500 runs in total), and the average of the 20 results was used for comparing the performance of ANN models. The most appropriate number of hidden nodes was identified through minimizing the negative log-likelihood statistics of the validation set. Because multiple responses (qL*, qa* and qb*) were predicted using the same ANN model, separate log-likelihoods were computed for each response (each color coordinate), and the overall log-likelihood for all the responses was calculated by adding the log-likelihoods of the individual responses [39]. The hyperbolic tangent (TanH) was used as the transfer function for the hidden layer of nodes, with automated, behind the scenes optimization of the penalty parameters [39]. Because the log-likelihood statistics indicated that the optimum number of nodes in the hidden layer was three, all subsequent analyses used the ANN model with two nodes (SR-S and Lb-A) in the input layer, three nodes in the hidden layer, and three nodes (qL*, qa* and qb*) in the output layer (Figure 1).

### 2.5. Calculating CMQ Coordinates from the Content of Pigments

Because the ANN model performed the best of the four models when three color coordinates were considered (Table 1), only this model was used in the subsequent analyses. To understand how CMQ color changes with varying levels of both pigments, the ANN model was applied to predict qL*, qa* and qb* coordinates for all possible combinations of SR-S (chlorophylls) and Lb-A (anthocyanins) values detected in tested plant species (Additional files 1 and 2 in Supplementary Materials). These models provide information about the gamut of qL*, qa*, qb*, qC* and *qh°* values that can be expected for each pigment individually, as well as for all their combinations. Correlations between the modeled and the observed CMQ coordinates were calculated using the Pearson correlation coefficient (*r*). Though the Pearson correlation coefficient provides good information about the closeness of data to the best-fitting line, it does not take into consideration how the best-fitting line conforms to the identity line. Therefore, Lin’s concordance coefficient (*ρ_c_*), which combines both the closeness of the data and the conformance to the identity line [40], was also calculated.

### 2.6. Testing the ANN Model on Other Plant Species

Though the CMQ model was developed using lettuce leaves, for its broad application on herbaceous leaves it is desirable that the model also provides accurate estimates of quasi-color coordinates for other plant species. To investigate how well the color model developed on lettuce leaves works on other plant species, comparisons between the predicted and the observed coordinates were performed on data from the ancillary dataset (Additional file 2 in Supplementary Materials). This dataset includes data from leafy vegetables and other, mostly herbaceous plant species with thin leaves quite similar to lettuce. The ancillary dataset is not intended to provide exhaustive information about leaf color for all included species; rather, it holds data about the common leaf colors of these species. This dataset also contains a few samples that were noticeably very different from healthy lettuce leaves, such as yellow-, almost white-, and orange-colored leaves, thick leaves, colored flower bracts, and white flower petals. These extra samples were included in the dataset to assess the limitations of the model outside of its original scope. To evaluate the performance of the model on samples in the ancillary dataset, differences between the modeled and the observed coordinates, as well as overall difference in color ($∆E=\sqrt{∆L^{2}+∆a^{2}+∆b^{2}}$), were calculated.

### 2.7. Statistical Analyses

All statistical analyses were performed using JMP software v. 14.2.0 (SAS Institute, Cary, NC, USA) and Microsoft Excel for Mac v. 16.16.10 (Microsoft, Redmond, WA, USA).

## 3. Results and Discussion

A total of 733 samples from 86 plant species were analyzed for the content of chlorophylls and anthocyanins, and their color. The primary dataset comprised 604 leaf samples from lettuce only, while the more diverse, ancillary dataset comprised 129 samples from 86 species. The primary dataset was used to test all models and to select the best model for predicting CMQ coordinates, while the ancillary dataset was employed to confirm that the model developed on lettuce leaves also provides accurate results for herbaceous leaves from other plant species.

### 3.1. Content of Pigments in Lettuce Leaves, Leaf Color and Comparison of Models to Predict CMQ Coordinates

The content of chlorophylls in the analyzed lettuce leaves ranged from 0 to 8.6 SR-S units, with the mean value of 5.8 (Additional file 1 in Supplementary Materials). The content of anthocyanins was in the range from 0.1 to 7.8 Lb-A units, with the mean of 3.6. The qL* ranged from 25.5 to 86 (mean = 41), qa* from −19.7 to 29.1 (mean = −2.8) and qb* from −4.4 to 43.8 (mean = 15.7). Significant differences were observed between colors on the adaxial and abaxial surfaces of the same leaves, with lower values of L*, b*, C* and *h°*, but higher values of a*, on adaxial surfaces. On average, the difference in color of the two surfaces was Δ*E_DB_* = 10.3 (Additional file 3 in Supplementary Materials). The largest difference (Δ*E_DB_* = 46.4) was detected on lettuce leaves with a very dark red adaxial surface ($L_{D}^{*}=26.2$,$\;a_{D}^{*}=8$, $b_{D}^{*}=-9.9,\;C_{D}^{*}=12.7,\;h_{D}^{{}^{\circ}}=309$) but a green abaxial surface ($L_{B}^{*}$ = 44,$\;a_{B}^{*}=-12$, $b_{B}^{*}$ = 28, $C_{B}^{*}=30.5,\;h_{B}^{{}^{\circ}}=113$). These data indicate that associating the content of pigments with color on only one of the leaf surfaces does not provide complete information and is not suitable for modeling.

The performance of the ANN model with three hidden layers was compared to the use of three parametric models on the primary dataset (604 lettuce leaves). On the testing set (124 samples), the highest *R*^2^ and the lowest RMSE values for qa* (0.979 and 1.08, respectively) and qb* (0.971 and 1.91, respectively) were achieved using the ANN model (Table 1). When the values for qL* coordinate were considered, the response surface model slightly outperformed the ANN model (*R*^2^ of 0.982 versus 0.981, and RMSE of 1.69 versus 1.71). Overall, the best match between the modeled and observed coordinates of the CMQ were found using the ANN model, followed by the response surface and quadratic polynomial models. A highly similar pattern was observed in data from the training set (480 samples). These results indicate that the ANN model provides the most accurate estimates of CMQ coordinates based on the concentrations of chlorophylls and anthocyanins in lettuce leaves. Conceivably, this outcome is a result of the ANN model being able to better capture the degree of spectral inference between pigments than the three tested parametric approaches. The superiority of ANN over response surface in capturing non-linear behavior was previously reported in several other [25,29,41,42], but not all [43], evaluated systems.

### 3.2. Modeling the Gamut of CMQ Coordinates

**Figure 2:**
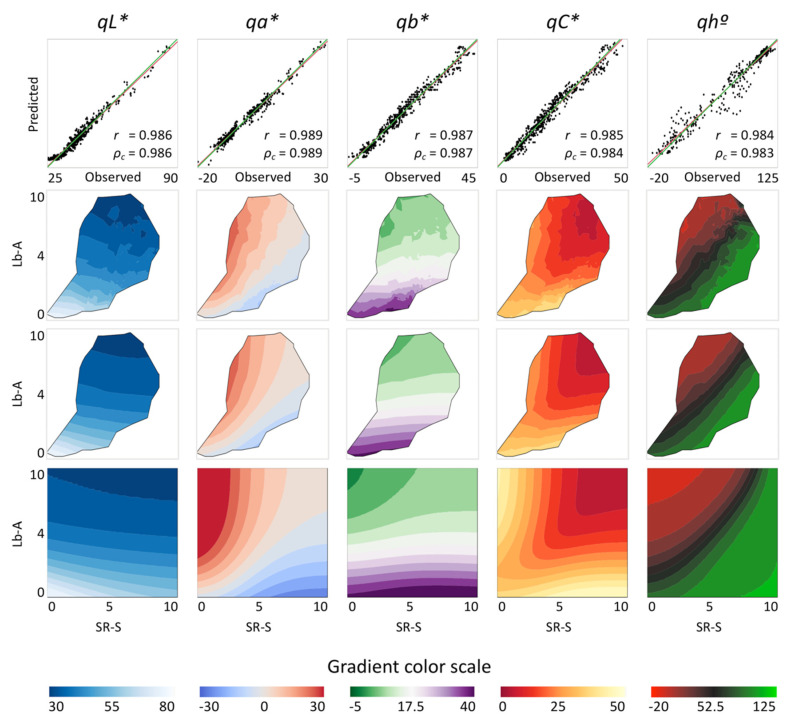
Observed and predicted coordinates of qL*, qa*, qb*, qC* and *qh°* for conceptual midpoint quasi-color (CMQ) assessed on 604 samples of cultivated lettuce. Top row shows linear correlations between observed and predicted values. Predicted values were calculated from contents of chlorophylls (SR-S values) and anthocyanins (Lb-A values) using an artificial neural network (ANN). The red lines indicate the best linear fit between observed and predicted values, while the green lines are identity lines (x = y). All correlation coefficients were highly significant (*p* < 0.0001, *n* = 604). Scales of *x*-axis and *y*-axis are identical for individual CMQ coordinates, thus for clarity of the figure only the range on the *x*-axes are indicated. Rows two to four show 3D presentations of values for each CMQ coordinate. Row two shows observed values from 604 lettuce samples, row three shows predicted values using an ANN, and row four shows predicted values for the extrapolated range of pigments (those combinations not present in the original samples). Note that rows two and three show values for the range of chlorophylls and anthocyanins detected in lettuce leaves only, while row four shows also values extrapolated outside of this range, but found in other tested plant species. Color gradients for CMQ coordinates in rows two to four are indicated at the bottom of the figure.

Because the ANN model performed better than the parametric models, only this model was used in the subsequent analyses. The analysis of CMQ coordinates in the data from 604 lettuce leaves (primary dataset, Additional file 1 in Supplementary Materials) revealed a strong association between the contents of plant pigments and qL*, qa* and qb*. The linear correlation between expected and observed values was 0.986 for qL*, 0.989 for qa* and 0.987 for qb* (Figure 2, top row). Chromaticity and hue angle also showed a highly significant (*p* < 0.0001, *n* = 604) linear correlation between expected and observed values (*r* = 0.985 for qC*, and 0.984 for *qh°*). The Lin’s concordance coefficient ranged from *ρ_c_* 0.983 to 0.989, demonstrating not only that correlations were high, but that the values were close to identity lines.

**Figure 3:**
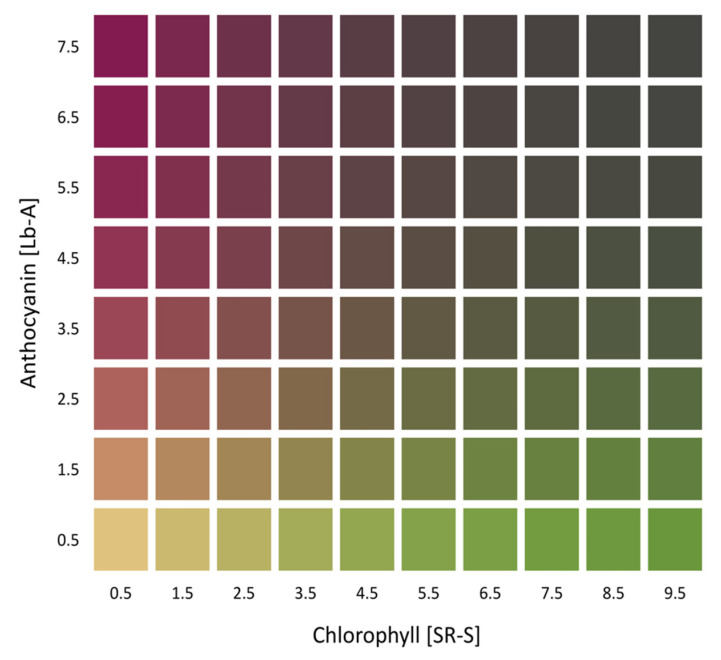
Predictive modeling of a leaf conceptual midpoint quasi-color (CMQ) from the content of chlorophylls and anthocyanins. CMQ values were predicted using the artificial neural network (ANN) approach. The results are shown for the approximate range of pigments content that was found in the analyzed set of leaves (Additional files 1 and 2 in Supplementary Materials). When the colorations of a leaf’s adaxial and abaxial surfaces are identical, the qL*, qa* and qb* coordinates predicted for the CMQ are the same as the L*, a* and b* coordinates predicted for each of the two leaf surfaces.

The additional rows in Figure 2 show 3D presentations of observed CMQ values from 604 lettuce samples (second row), predicted CMQ values using the ANN model (third row), and predicted CMQ values for the range of pigments found in all tested plants (fourth row). This range of pigments includes also hypothetical combinations of chlorophylls and anthocyanins that were not actually detected in the analyzed samples. Modeled values of qL* decreased with the increasing content of both pigments, while the qa* and qb* values were affected more by the relative content of chlorophylls and anthocyanins in leaves (Figure 2). The values of all three coordinates, however, were affected more by Lb-A than SR-S, with the relative impact of the independent variables on qL* being 90.6% and 5.6%, on qa* 60.4% and 36.1%, and on qb* 83.6% and 12.8%, respectively. The highest vividness of quasi-colors (largest qC*) was detected in leaves with high contents of either pigment alone. If both pigments were present in the leaf together, particularly at very high contents, the quasi-color became duller (Figure 3, upper right corner). When leaves contained only a high content of anthocyanins, the modeled hue angle was in the purple–red range (~340°) (Figure 3, upper left corner), while for leaves that contained only a high content of chlorophylls, the modeled hue angle was in the green range (~125°) (Figure 3, lower right corner). For leaves with identical coloration on adaxial and abaxial surfaces, the qL*, qa* and qb* coordinates predicted for the CMQ are the same as the L*, a* and b* coordinates predicted for each of the two leaf surfaces. The ANN formulas for the CMQ model are:

qL* = 76.8278 − 23.3637 * H1 + 0.6636 * H2 −24.9085 * H3 (1)

qa* = −13.0326 + 24.3295 * H1+ 34.7418 * H2 + 22.0398 * H3 (2)

qb* = 28.5284 − 34.0210 * H1 − 5.4716 * H2 + 0.7791 * H3 (3)

where,

H1 = Tanh [0.5 * (−1.0617 + 0.0311 * SR-S + 0.6420 * Lb-A)](4)

H2 = Tanh [0.5 * (1.4045 − 0.5334 * SR-S − 0.0241 * Lb-A)](5)

H3 = Tanh [0.5 * (−0.1777 + 0.3507 * SR-S + 0.9401 * Lb-A)](6)

**Figure 4:**
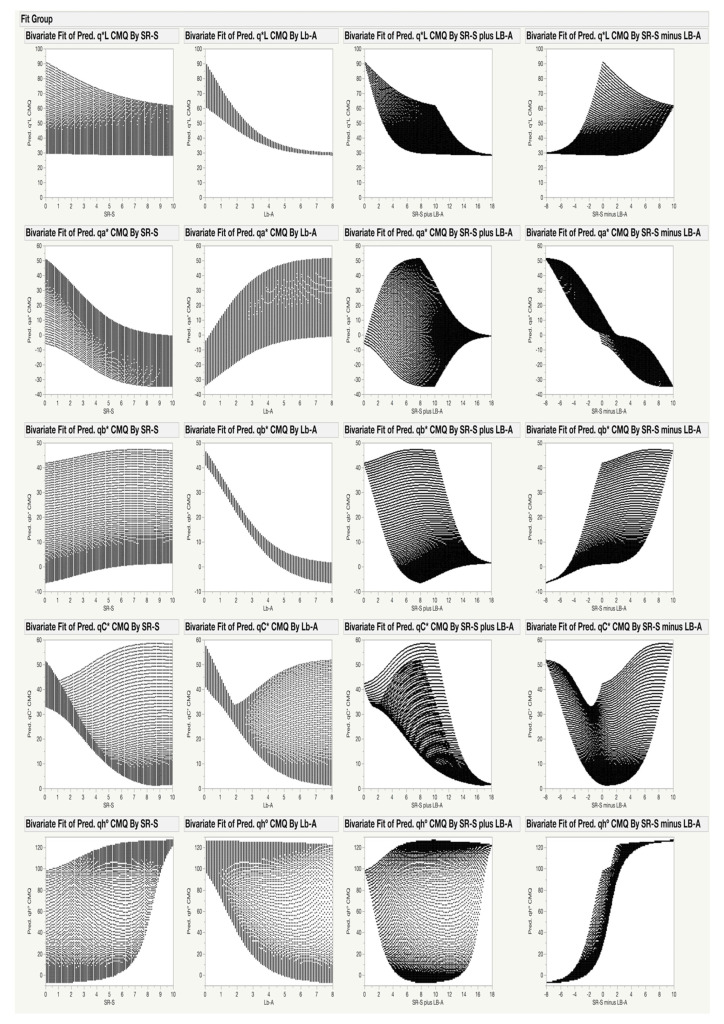
Visualization of the relationship between the predicted values of qL*, qa*, qb*, qC* and *qh°* coordinates for CMQ, and the range of chlorophylls and anthocyanins content detected in analyzed plants. Predicted values of qL*, qa*, qb*, qC* and *qh°* coordinates (rows) were plotted against values of SR-S, Lb-A, SR-S plus Lb-A, and SR-S minus Lb-A (columns), in order to visualize the relationship between pigments content and CMQ coordinates. These graphs show how each of the pigments and their combinations affect qL*, qa*, qb*, qC* and *qh°* values. For example, when the SR-S value is equal to 5, the predicted qL* values will be approximately in the range of 30–70 (upper left panel).

The gamut of possible qL*, qa*, qb*, qC* and *qh°* values, and their distribution for each pigment individually, but also for their combination, is shown in Figure 4. For example, the values of qb* (Figure 4, third row) for the CMQ can be in the wide range of ~0 to ~45 when the SR-S is 5.0. However, the range of possible qb* values for the CMQ is much narrower for any particular content of Lb-A; e.g., only from ~4 to ~10 for Lb-A of 4.0. The figure also shows how the values of each coordinate change when the combined content of both pigments increases (‘plus values’ in the third column), or when the difference in the content of pigments changes (‘minus values’ in the fourth column). Based on the ANN model, the only possible values of qL*, qa*, qb*, qC* and *qh°* for the CMQ are those shown in Figure 4.

### 3.3. Performance of the ANN Model on Herbaceous Leaves

Lettuce was used to develop the ANN model because of the high variability in its leaf color and concentration of pigments. However, for a broad application on herbaceous leaves, the model also needs to provide reliable results for other plant species with similar leaf characteristics. To test how the CMQ model that was developed for cultivated lettuce applies to other plants, further assessments were performed on 129 additional samples from other species and cultivars that showed a wider range in their contents of both chlorophylls (SR-S from 0 to 9.7) and anthocyanins (Lb-A from 0 to 8.0) (ancillary dataset, Additional file 2 in Supplementary Materials) than was observed for the analyzed lettuces (primary dataset, Additional file 1 in Supplementary Materials). The differences between the predicted and observed values for samples in this dataset were larger than for samples in the primary dataset. The values of Δ*qL* ranged from −11.6 to 16.0, of Δ*qa* from −5.5 to 15.0, and of Δ*qb* from −36.1 to 28.8 (Additional files 2 and 4 in Supplementary Materials). When all the coordinates were considered, the largest differences (Δ*qE*) were found for samples with almost no chlorophyll when they visually appeared white (flower petal of daisy Δ*qE* = 36.4), bright red (bract of poinsettia Δ*qE* = 33.7), intensive yellow (dying leaf of bear’s breech Δ*qE* = 27.1) or were thick and/or dense (camellia Δ*qE* = 17.5) (Additional files 2 and 4 in Supplementary Materials). The model worked well for all tested leafy vegetables (chard, cultivated endive, common dandelion, Lewiston cornsalad, pak choi, radicchio, rocket salad, spinach, tender green and treviso) regardless of their color, and also on other plant species with comparable leaf characteristics. The test indicates, however, that the ANN-based CMQ model, which was developed on thin, photosynthetically active, herbaceous leaves of lettuce, may be less reliable for predicting the quasi-color values of leaves with substantially different structures, thicknesses, dry masses, pHs (which can change anthocyanin color) and pigment compositions, or for leaves that are covered with wax, trichomes or other features modifying the visual appearance of their color.

From the set of tested samples, the CMQ model predicted the darkest quasi-color (lowest qL* value) for mondo grass (*Ophiopogon planiscapus* Nakai) cv. Nigrescens, which contains large quantities of both chlorophylls (SR-S of 9.7) and anthocyanins (Lb-A of 7.5). The modeling results (qL* = 28.7, qa* = −0.9 and qb* = 1.7) matched well with the empirical values found in this (qL* = 25.8, qa* = −1.6 and qb* = 3.2) (Additional file 2 in Supplementary Materials) and earlier [44] studies (qL* = 29, qa* = −1.9 and qb* = 3.3 were calculated from the values of L*, C* and *h°* reported for both leaf surfaces). It was previously shown that the blackish color of this cultivar was caused by abundant contents of both chlorophylls and anthocyanins, but not carotenoids [44]. Results of the present study also show that estimating the content of chlorophylls and anthocyanins from the visual appearance of leaves may be problematic when both pigments are present in the samples simultaneously, because the perceived ‘greenness’ or ‘redness’ of a leaf is affected not only by the content, but also by the ratio of the two pigments. For example, when both anthocyanins and chlorophylls were present in lettuce leaves in detectable quantities, no correlation was found between the chlorophyll content and values of a* or b* [45]. Such results accentuate the conclusions of the present study, regarding the combined effect of both pigments on the CIELab color coordinates of a leaf (Figure 2, Figure 3 and Figure 4). In addition, modeling of the CMQ coordinates shows that when both chlorophylls and anthocyanins are present in the same leaf tissue, a correlation between the content of chlorophylls and the qL*, qa* or qb* coordinates (or derived qC*, *qh*°) may not be detected because of the spectral interference between the two pigments. However, there appears to be a strong relationship between the content of anthocyanins and color coordinates (Figure 4, second column), particularly with qL* and qb*, though even these may be affected to a certain extent by the presence of chlorophylls.

### 3.4. Dorsiventral Distribution of Pigments

Current analysis and modeling show that highly different colors of leaves may result from similar contents of pigments but their different dorsiventral distribution. For example, leaf samples LS-385 and LS-101 (Additional file 1 in Supplementary Materials) have similar contents of chlorophylls (SR-S of 5.4 for both) and anthocyanins (Lb-A of 3.7 and 3.9, respectively). Therefore, they also have similar qL* (37.1 and 36.1), qa* (1.4 and 2.3) and qb* (11.5 and 10.2) coordinates, predicted for CMQ. However, while LS-385 has a fairly similar color on both leaf surfaces (adaxial L_D_* = 33.9, a_D_* = 1.7 and b_D_* = 8.3; abaxial L_B_* = 37.0, a_B_* = 2.6, b_B_* = 10.1; Δ*E_DB_* = 3.7), the color of the leaf surfaces is highly different for LS-101 (adaxial L_D_* = 23.1, a_D_* = 8.1 and b_D_* = 5.2; abaxial L_B_* = 50.3, a_B_* = −4.1, b_B_* = 16.3; Δ*E_DB_* = 31.8). Leaf color modeling approaches that consider only one, usually adaxial, surface of the leaves, or assume that both surfaces have a similar color, would indicate that the color of either LS-385 or LS-101 is in discrepancy with the content of pigments. The CMQ approach allows, however, for such differences in colors. Assuming that the actual color for one of the leaf surfaces (e.g., adaxial) can be obtained, it is possible to estimate the unknown color of the abaxial surface by subtracting the color coordinates of the adaxial surface from the CMQ coordinates (multiplied by two, because they correspond to the mean of both surfaces), modeled from the content of pigments. In this example, the expected coordinates for LS-385 (qL_B_* = 40.2, qa_B_* = 1.0 and qb_B_* = 14.6) and LS-101 (qL_B_* = 49.2, qa_B_* = −3.6 and qb_B_* = 15.2) are in the range of the actual values measured on the abaxial surfaces, confirming that highly different colors may be observed on leaves with similar contents of pigments but different dorsiventral distributions. Such dorsiventral distribution of pigments needs to be taken into consideration when modeling [6,7,8,9,10] or monitoring [46,47] leaf colors and their changes, particularly as regards plant species or cultivars with high contents of both pigments.

### 3.5. Application of CMQ in Plant Research

Due to spectral inference between pigments [4], the concentration of chlorophylls and anthocyanins cannot be accurately estimated from fruit [11] or leaf [4] color when they co-occur within the same tissue in high enough quantities. A limited approximation of the concentration range for anthocyanins can be deduced from the CMQ coordinates calculated from the abaxial and adaxial leaf colors (Figure 4, second column). However, no reliable estimate is possible for most of the concentrations of chlorophylls (Figure 4, first column), because chlorophylls affect the change in color coordinates relatively less significantly than anthocyanins do (~6:91 ratio for qL*, ~36:60 ratio for qa* and ~13:84 ration for qb*).

The CMQ approach could be used to model virtual [6,7,8,9,10] colors of leaves [12] when studying the mechanism of color change [13,48]. The approach could also be used to model ‘customized’ leaf colors [49] in environments where both leaf surfaces are not observable. For example, when the abaxial color of prostrate leaves cannot be seen on plants grown in controlled-environment agriculture (indoor farms), but knowing that color is important to the industry, concentrations of pigments for a particular cultivar could be modeled [10] from nutrition [50], temperature [19], light [51,52] and other environmental data, while the adaxial color of leaves can be measured with overhead sensors. CMQ coordinates (calculated from modeled concentrations of pigments using formulas provided in this study) can then be applied, together with adaxial color data, to estimate abaxial leaf color.

## 4. Conclusions

This work provides the proof of concept for the use of CMQ in plant research. The ANN model outperformed the full factorial, quadratic polynomial and response surface models for predicting the qa* and qb* coordinates of CMQ, and was only marginally worse than the response surface model in predicting qL* values. These results suggest that the ANN model surpasses the parametric models in capturing spectral inference between pigments. Thus, the ANN-based CMQ model can be used to test the distribution of color in the bifacial leaves of lettuce and other leafy vegetables, as well as its diversity and association with pigments. This approach may be particularly helpful when modeling the color of leaves with large dorsiventral differences. In such leaves, combining the color coordinates from both surfaces allows for finer association with the content of chlorophylls and anthocyanins than does use of color data from a single surface only. However, it may be necessary to develop specific models for other types of leaves that do not conform to the lettuce-based model. Though CMQ coordinates per se do not indicate the actual leaf color, these coordinates match well with the content of pigments in leaf tissue. CMQ coordinates could thus be used, for example, for estimating L*, a* and b* on an unobservable leaf surface when the content of chlorophylls and anthocyanins is known, and the color coordinates for one of the surfaces could be measured. For plants with similar coloration on both leaf surfaces, the CMQ model could be used to directly predict their color from their content of chlorophylls and anthocyanins. When the colorations of a leaf’s adaxial and abaxial surfaces are identical, the qL*, qa* and qb* coordinates predicted for CMQ are the same as L*, a* and b* coordinates predicted for each of the two leaf surfaces.

## Figures and Tables

**Table 1:** Performance of parametric and empirical models in predicting coordinates of conceptual midpoint quasi-color (CMQ).

Model	Training Set (480 Samples)						Testing Set (124 Samples)					
	*R* ^2^			RMSE			*R* ^2^			RMSE		
	qL*	qa*	qb*	qL*	qa*	qb*	qL*	qa*	qb*	qL*	qa*	qb*
Full factorial	0.945	0.844	0.917	2.37	2.72	3.04	0.963	0.857	0.913	2.31	2.65	3.31
Quadratic polynomial	0.962	0.957	0.971	2.00	1.73	1.84	0.978	0.945	0.965	1.83	1.77	2.13
Response surface	0.970	0.964	0.972	1.76	1.58	1.82	0.982	0.950	0.965	1.69	1.66	2.15
Artificial neural network	0.969	0.979	0.974	1.81	1.23	1.71	0.981	0.979	0.971	1.71	1.08	1.91

*R*^2^: coefficient of determination. RMSE: root mean square error. qL*, qa*, qb*: CIELab color space coordinates for CMQ.

## Supplementary Materials

The following are available online at https://www.mdpi.com/1424-8220/20/14/3938/s1, Dataset S1: The list of 604 lettuce samples that were used to develop the ANN model of leaf color (primary dataset). Dataset S2: The list of 129 samples that were used to assess performance of the lettuce-based model of leaf color developed through ANN approach (ancillary dataset). Figure S3: Distribution of differences between color coordinates measured on adaxial and abaxial surfaces of 604 lettuce leaves. Figure S4: Distribution of differences between predicted and observed CMQ coordinates for 129 samples.
