# Supplementary figures and images for: Predictive Modeling of a Leaf Conceptual Midpoint Quasi-Color (CMQ) Using an Artificial Neural Network
Source: Sensors (Basel). 2020 Jul 15;20(14):3938. doi: 10.3390/s20143938 (PMC7412459; doi:10.3390/s20143938)

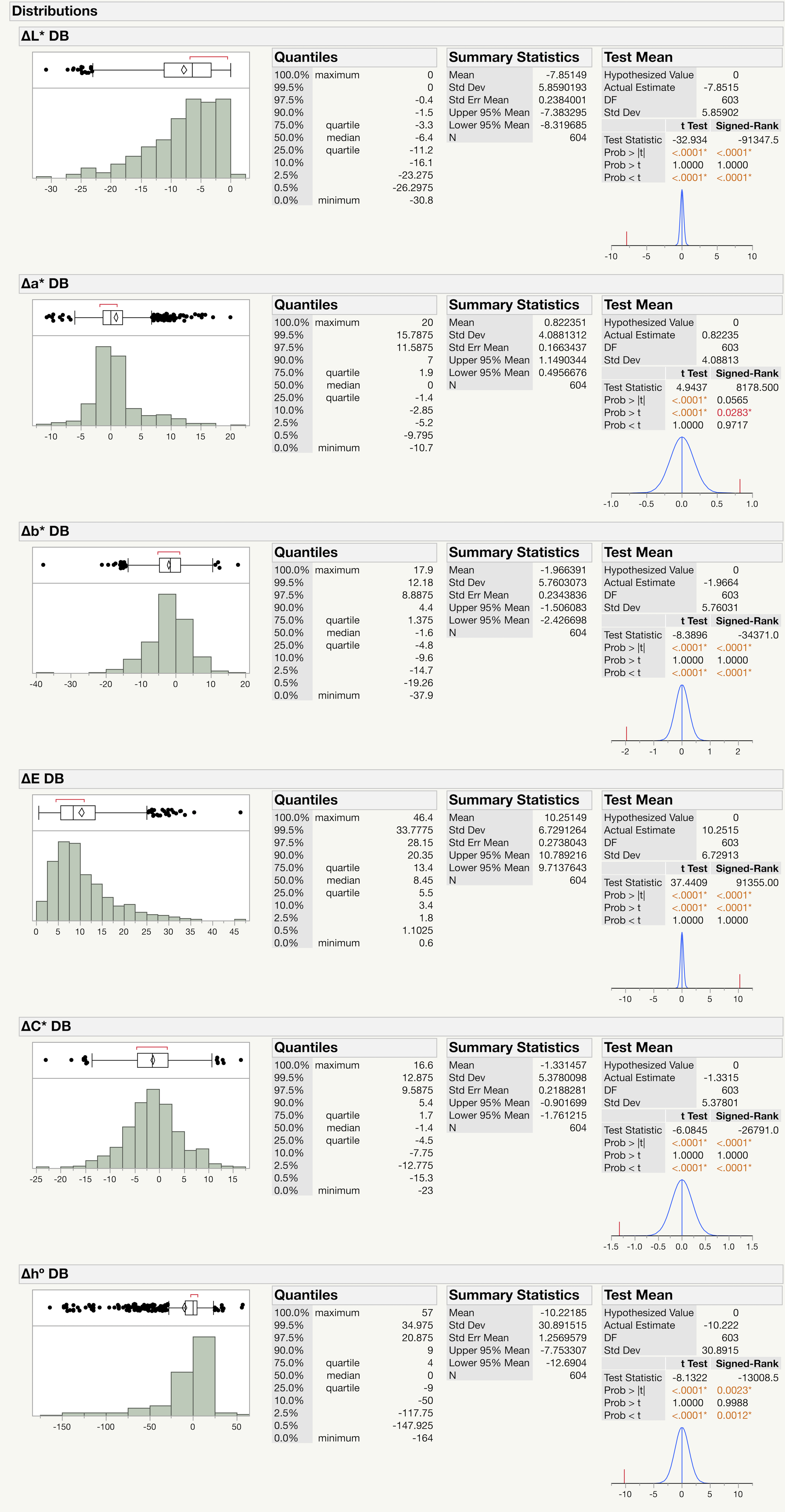

Supplement: Supplementary file 1 [file sensors-20-03938-s001.zip › Figure S3.png]
